# On-chip detection of gel transition temperature using a novel micro-thermomechanical method

**DOI:** 10.1371/journal.pone.0183492

**Published:** 2017-08-17

**Authors:** Tsenguun Byambadorj, Erfan Dashtimoghadam, Mohamadali Malakoutian, Benyamin Davaji, Lobat Tayebi, James E. Richie, Chung Hoon Lee

**Affiliations:** 1 Department of Electrical and Computer Engineering, Marquette University, Milwaukee, United States of America; 2 School of Dentistry, Marquette University, Milwaukee, United States of America; 3 School of Electrical and Computer Engineering, Cornell University, Ithaca, NY, United States of America; University of South Carolina, UNITED STATES

## Abstract

We present a new thermomechanical method and a platform to measure the phase transition temperature at microscale. A thin film metal sensor on a membrane simultaneously measures both temperature and mechanical strain of the sample during heating and cooling cycles. This thermomechanical principle of operation is described in detail. Physical hydrogel samples are prepared as a disc-shaped gels (200 *μ*m thick and 1 mm diameter) and placed between an on-chip heater and sensor devices. The sol-gel transition temperature of gelatin solution at various concentrations, used as a model physical hydrogel, shows less than 3% deviation from in-depth rheological results. The developed thermomechanical methodology is promising for precise characterization of phase transition temperature of thermogels at microscale.

## Introduction

In biomedical and pharmaceutical applications, thermosensitive hydrogel systems are widely used to deliver various bioactive agents via a thermally triggered transition as the physical form changes from solution to gel [1–4]. The thermally triggered transition can be specified as the gelation temperature, which depends on various factors such as the rate of temperature change, the concentration, and the chemical and physical structures of the hydrogel [4, 5]. The gelation temperature is typically measured with a bulk sample, which requires slow heating to achieve a uniform temperature distribution across the sample [6]. Because the polymer chains in the physical hydrogel systems have steady association and disassociation with the heating rate, the gelation time is inevitably longer for a bulk sample [7] and dehydration may occur. At microscale, on the other hand, a higher heating rate can be applied to the sample because the thermal mass of the sample is orders of magnitude lower than that of a bulk sample. Furthermore, the temperature across the microscale sample is more uniform due to the minimal thermal loss to the environment. Therefore, the gelation temperature could be different at microscale from that at macroscale. A precise measurement of the gelation temperature for thermo-responsive hydrogels is required for applications such as drug delivery, tissue engineering and cell encapsulation as it defines the performance of the designed delivery systems [8–11].

A well-defined method measuring the precise gelation temperature of hydrogels at microscale could lead to the development of carrier gels with sol-gel transition at physiological temperature in various biomedical applications including delivery matrices for therapeutic compounds, 3D-scaffolds in regenerative medicine, cell encapsulation microenvironments [12–16].

The gelation temperature is commonly measured by rheology and differential scanning calorimetry (DSC) techniques, which are based on frequency-independent loss tangent from a multi-frequency plot versus temperature and enthalpy change, respectively. The sample size required for these methods is typically more than several hundred microliters for rheology and around 10 *mg* for DSC measurements. In fact, the measurement parameters are undetectable at microscale in these techniques. Moreover, in the case of rheological approach, determination of the phase transition temperature is discrete, which means rheological parameters are typically measured at stepwise equilibrated temperatures [17, 18]. Furthermore, ensuring a hermetic seal of the microscale sample to avoid dehydration is a challenge during the slow heating and cooling processes [19].

For a microscale sample, highly sensitive micromachined calorimeters have been used to measure thermal properties [20–22]. However, most micromachined calorimeters use closed chambers or microfluidic channels that are incompatible with gel samples [21, 22].

In this paper, we present a new thermomechanical method and a platform to detect the gelation temperature of hydrogels which have undetectably low enthalpy change at microscale. When a sample goes under thermally triggered sol-gel transition, the small change in volume causes stress on a SiN membrane, where a thin film metal temperature/strain sensor is integrated. The sensor continuously measures the sample temperature and detects the mechanical stress the sample exerts at the sol-gel transition. The platform can be used to measure the phase transition temperature of various other solid, gel, and liquid samples.

## Principle of operation

### Device fabrication and characterization

**Fig 1:**
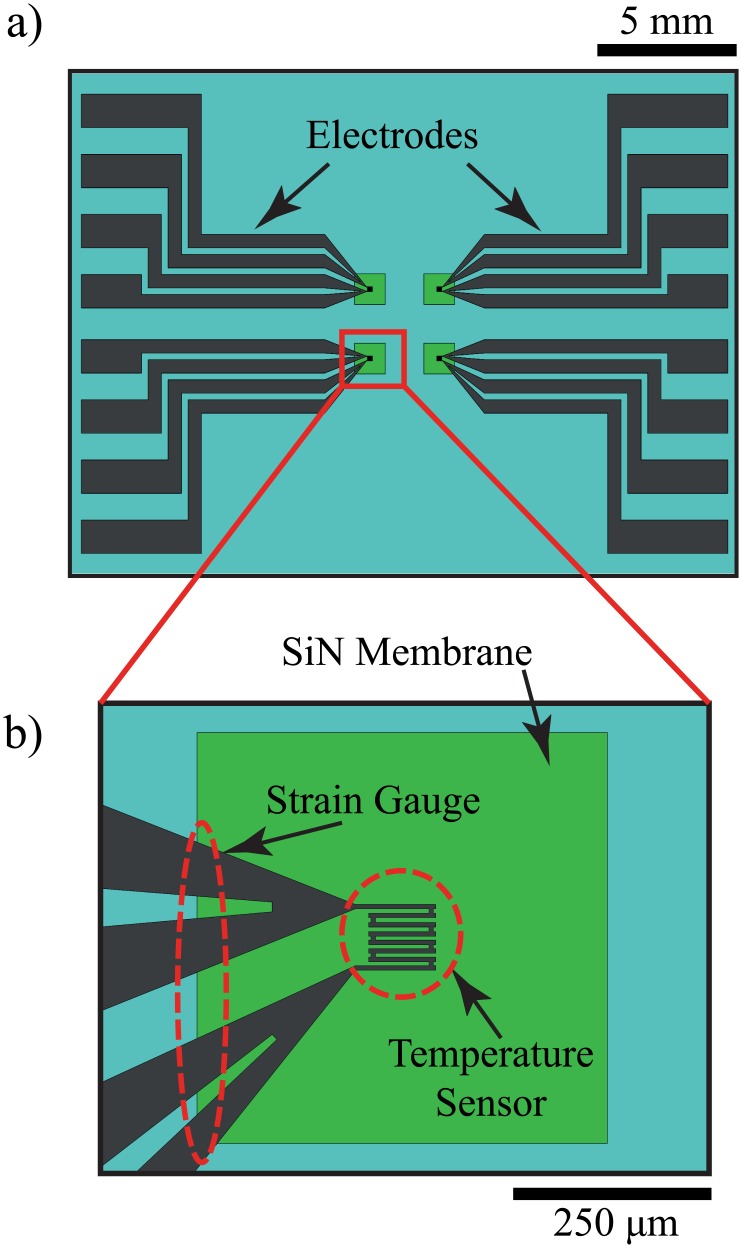
The top view of the on-chip heater or sensor #1 devices. The chip contains 4 identical resistive elements with 4 electrodes designated for a 4-wire measurement. The resistive element is a temperature sensitive (*α* = 2.5 × 10^−3^ °*C*^−1^) 40 *nm* nickel thin film for the sensor or nickel silicide on 500 *nm* polysilicon for the heater. Both are identically patterned on 500 *nm* thick silicon nitride membranes. (a) An overview of 2 *cm* by 1.5 *cm* chip device. (b) Close-up view of the resistive element pattern on a 500 *μm* by 500 *μm* membrane.

The micro-thermomechanical calorimeter system consists of two thermal elements, a sensor and a heater, in face-to-face sandwich configuration loaded with a sample in between. As shown in Fig 1, both the sensor (Ni) and the heater (polysilicon) are patterned as a serpentine shape on a 500 *nm* thick SiN membrane. The device fabrication process is reported elsewhere [23]. The heater and the sensor are electrically isolated by Kapton tape (50 *μm* thick) and an *Al*_2_*O*_3_ film (10 *nm* thick), respectively.

The sensor is a 40 *nm* thick nickel thin film (thermally evaporated) resistive temperature detector (RTD) integrated on the SiN membrane (Fig 1). The sensor is sensitive to both temperature (as an RTD) and mechanical stress (as a piezoresistor). The piezoresistor can be optimized further for higher sensitivity. To improve the sensitivity, a serpentine shape resistor can be integrated separately across the edge of the membrane. In order to measure temperature and strain independently, the distance between the temperature sensor and the strain gauge should be sufficiently large. The separate configuration requires more electrodes and another Source/Meter Unit (SMU).

The heater is a 500 *nm* thick LPCVD phosphorous-doped polysilicon with the same pattern as the sensor. The heater can withstand high temperature (> 200°*C*) and last for an extensive period of operation. To protect the device from oxidation and improve the electrical conductivity, the polysilicon is annealed with a thermally evaporated thin nickel film at 600°*C* for 30 minutes [24]. After the metal silicidation, the resistance of the heater decreases from 20 *kΩ* to 3-5 *kΩ*. The heater is covered by 50 *μm* thick Kapton tape (glass transition temperature of 400°*C*) [25] to prevent electrical cross-talk to the sensor and direct physical contact to the sample. Heat is applied to a sample by applying a voltage pulse to the heater, which raises the sample temperature by Joule heating.

The principle of our thermomechanical measurement of the hydrogel transition temperature is the following. Pulsed heat is applied to the sample. The sensor continuously measures both temperature and strain simultaneously. Until the sample undergoes the phase transition, the sensor only shows a temperature change because there is no significant mechanical deformation. Therefore, the resistance change of the sensor is only due to the temperature change of the sample. The temperature profile follows the first order heat transfer [26]. At the phase transition, mechanical strain is applied to the sensor membrane due to the physical deformation of the sample. The mechanical strain causes a resistance change of the sensor in addition to the resistance change due to the temperature change. The additional resistance change deviates the resistance change profile of the sample from that of temperature alone. The deviation point of the resistance change from the temperature profile caused by the first order heat transfer indicates the phase transition temperature of the sample.

Two sensors are prepared to study the effect of membrane size on the sensitivity of the strain caused by the sample at the phase transition temperature. Sensor #1 is integrated on a 500 *μm* by 500 *μm* SiN membrane and sensor #2 on a 5 *mm* by 5 *mm* SiN membrane. The devices are housed in custom-made acrylic templates and their electrodes are electrically bonded by silver epoxy.

The strain is measured based on the resistance change of the piezoresistive nickel thin film integrated across the edge of the membrane [27]. From Kirchhoff-Love plate theory, the deflection and strain of a fixed square plate can be expressed as [28],

$$\begin{array}{c} w=\frac{k_{1}\cdot q\cdot a^{4}}{D} \end{array}$$ (1)

$$\begin{array}{c} \epsilon =\frac{k_{2}\cdot h\cdot q\cdot a^{2}}{D} \end{array}$$ (2)

where *w*(*m*) is the deflection, *q*(*N*/*m*^2^) is the distribution load, *a*(*m*) is the membrane length, *D*(*Pa*.*m*^3^) is the flexural rigidity, *h*(*m*) is the thickness, *k*_1_ and *k*_2_ are the constants, and *ϵ* is the strain. By calculating the load from Eq (1) and substitute in Eq (2), the linear relationship between the deflection and the strain can be expressed as,

$$\begin{array}{c} \epsilon =\frac{k_{3}\cdot h\cdot w}{a^{2}} \end{array}$$ (3)

**Fig 2:**
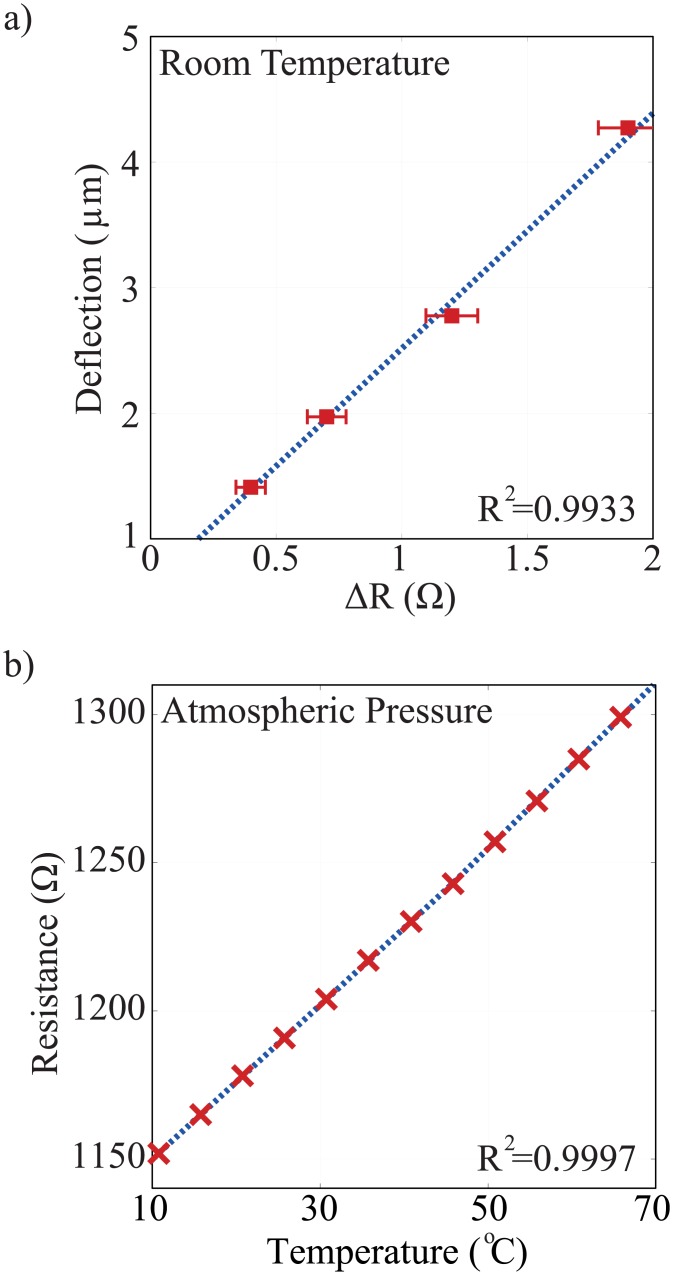
(a) The deflection-strain characteristics of the membrane. A linear relationship of the membrane deflection and resistance change is shown. The deflection is measured by a laser microscope, and the resistance is measured by a SMU. The resistance is proportional to the strain of the membrane. (b) RTD Linearity Measurement. The resistance of the sensor is measured as the temperature of the environment increases from 10°*C* to 70°*C* with a 2.5°*C*/*hour* increment. The resistance at the end of every increment is plotted against the temperature to find the linearity.

The resistance change as a function of strain can be expressed as [29],

$$\begin{array}{c} \Delta R=R_{0}\cdot GF\cdot \epsilon \end{array}$$ (4)

where Δ*R* (Ω) is the resistance change, *R*_0_ (Ω) is the resistance without deformation, and *GF* is the gauge factor. A linear relationship between the membrane deflection and resistance change is found. A variable negative load pressure controlled by a mass flow meter is applied to the membrane. Mass flow meter is connected to the closed PDMS chamber on the backside of the membrane using plastic tubing. The deflection is measured by a laser interferometry microscope (LEXT OLS4000) while the resistance change is measured by a Keithley 2600 SMU. The measured data is shown in Fig 2(a).

The resistance change of the sensor as a function of temperature up to the phase transition temperature can be expressed as,

$$\begin{array}{c} R_{T}=R_{0}\left(1+\alpha \left(T-T_{0}\right)\right) \end{array}$$ (5)

where *R*_*T*_ is the resistance at temperature *T*, *R*_0_ is the resistance at reference temperature *T*_0_, and *α* (°*C*^−1^) is the temperature coefficient of resistance (TCR). The TCR of the evaporated thin film RTD is measured with a temperature-controlled environment with 2.5°*C*/*hour* increment to allow stable temperature. The measurement shows a TCR of 2.5 × 10^−3^ °*C*^−1^ with a linear response (coefficient of determination *R*^2^ = 0.9997) in the measurement range (10-70°*C*) as shown in Fig 2(b). For bulk nickel, the TCR is 6.2 × 10^−3^ °*C*^−1^. The lower alpha of the thin film nickel sensor may be due to the granularity of the evaporated film and the effect of thickness [29, 30].

The resistance change of the sensor due to temperature and strain is measured by the 4-wire measurement using a SMU. The details of the fabrication process and resistance measurement method are presented elsewhere [20, 31].

### Sample preparation

Two materials are prepared for the thermomechanical phase transition temperature measurement: purified paraffin wax and gelatin as a model physical gelling system.

Purified paraffin wax with known melting temperature (Sigma-Aldrich 327204) is used to verify the micro-thermomechanical method for the phase transition measurement. The phase transition temperature results are compared with those of a commercially available differential scanning calorimeter (DSC). A paraffin wax is prepared in a 200 *μm* thick PDMS mold, resulting in disks with a 2.5 *mm* diameter and a 200 *μm* thickness. The thickness and volume of the samples are chosen based on the thermal time constant, which should be shorter than the pulse width of the heater [32].

The model physical gelling system used in this work is gelatin. Gelatin (Type A, 300 bloom from porcine skin) was purchased from Sigma-Aldrich and is dissolved in deionized water for various aqueous solutions with different weight concentrations (10, 15, and 20 wt%) to achieve various concentration induced gelation temperatures. A fresh PDMS mold is used to form hydrogel disks. A 1 *μl* gelatin solution is dropped in the PDMS mold by a micropipette and cooled into gel phase to form a thin disk with 2.5 *mm* diameter. The volume of both wax and hydrogel between the 500 *μm* by 500 *μm* membranes is 50 *nl*.

### Rheological measurement of sol-gel transition temperature

Oscillatory shear experiments are performed in a Malvern Kinexus pro+ rheometer using a cone-and-plate geometry, with a diameter of 40 mm and a cone angle of 4 degree. The measuring apparatus is equipped with a temperature unit, which provides an effective temperature control (±0.01°*C*) for an extended time over the studied temperature range. Strain amplitude of 1% is applied to minimize the perturbation of the network during the gel evolution process. Before the rheological measurements, the strain amplitude was checked to ensure the measurements are conducted within the linear viscoelastic regime and the dynamic storage modulus (G′) and loss modulus (G′′) to be independent of the strain amplitude. The gelation temperature of the gelatin formulations is determined by frequency-independent value of the loss tangent (tan*δ* = G′′/G′) obtained from a multi-frequency plot versus temperature. An alternative method is also employed to determine the gel point, which is based on crossover of the apparent viscoelastic exponents n′ and n′′ ($\text{G}\prime \;\sim \;\omega^{n^{\prime }}$, $\text{G}\prime \prime \;\sim \;\omega^{n^{\prime \prime }}$) calculated from the frequency dependence of G′ and G′′ at different temperatures [33, 34].

### Measurement method

**Fig 3:**
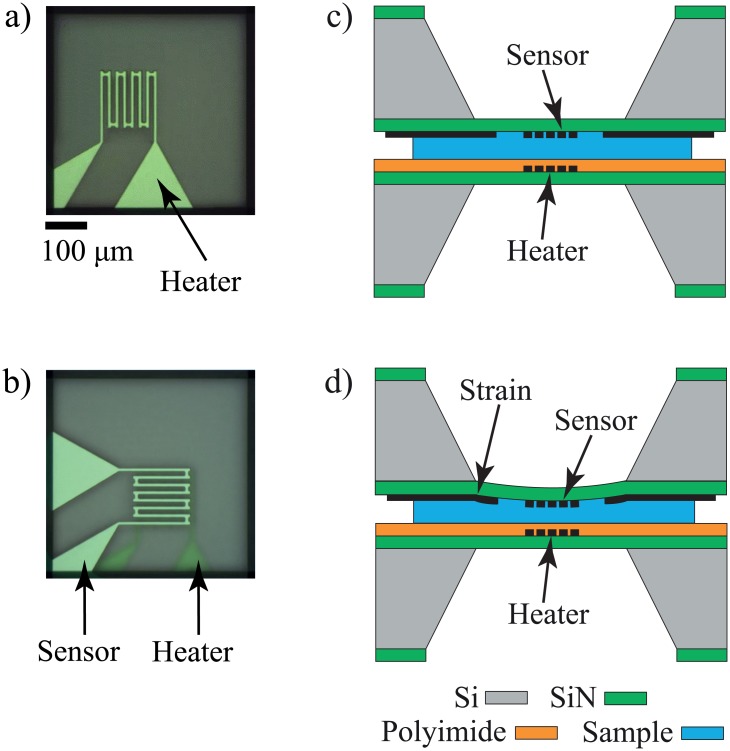
Schematic drawings of device and sample preparation for thermomechanical gelation measurement setup. (a) and (b) are top view of the setup, and (c) and (d) are the cross-sectional view of the setup. a) Top view of the heater device via optical microscope camera. b) The sandwich configuration of the sensor and heater with sample in between. The transparency of the membrane allows optical alignment of the sensor over the heater device. c) Cross-sectional view of the measurement configuration, where the heater is coated with polyimide film while sensor is plain. d) Membrane deflection due to the stress exerted by volumetric change of sample during the phase transition.

The phase transition measurement is performed by placing the sample between the heater and sensor as shown in Fig 3. A voltage pulse is applied to the heater using Keithley 2400 SMU. Then, the temperature of the sample is monitored by measuring the resistance of the sensor. The sensor resistance is measured using a 4-wire configuration (Keithley 2600 SMU as a voltage pulse and Stanford current amplifier as a current meter). The heater (the bottom device) sits on a spring-loaded XY stage that can be pressed down as the sensor (the top device) is brought down to make a physical contact. The sensor is brought to the heater by a computer-controlled XYZ stage.

Fig 3(a) shows the top view of the heater. The sample is loaded between the heater and sensor membranes in a sandwich configuration as shown in Fig 3(b). The sensor is aligned with the heater by monitoring the backside of the sensor via a microscope camera. The alignment can be precisely done because the SiN membrane is transparent while the metal sensor and silicide polysilicon are distinctly visible as shown in Fig 3(c).

In our thermomechanical method, the phase transition of gelatin hydrogel is detected when the SiN membrane of sensor is deflected due to the physical deformation of the sample at the transition temperature. The sensor monitors both the temperature change and strain when the sample is heated past the gelation point and naturally cooled. At the gelation temperature, mechanical stress due to a physical deformation of the hydrogel is applied to the sensor membrane as shown in Fig 3(d), resulting in an abrupt change of resistance. The displacement of the heater membrane due to the stress from the hydrogel is much smaller than that of the sensor membrane because of the Kapton tape on the heater. The Kapton tape is placed on the heater membrane for electrical isolation and to make the heater membrane stiffer than the sensor membrane.

The distinct resistance change at the transition temperature is due to the membrane strain. The enthalpy change of the microscale hydrogel is too low to be detected by temperature changes at the transition temperature. This is confirmed by the experiments with no measurable resistance change when the sensor membrane is made stiffer by adding a 25 *μm* thick adhesive polyimide film.

## Results

### Method verification

**Fig 4:**
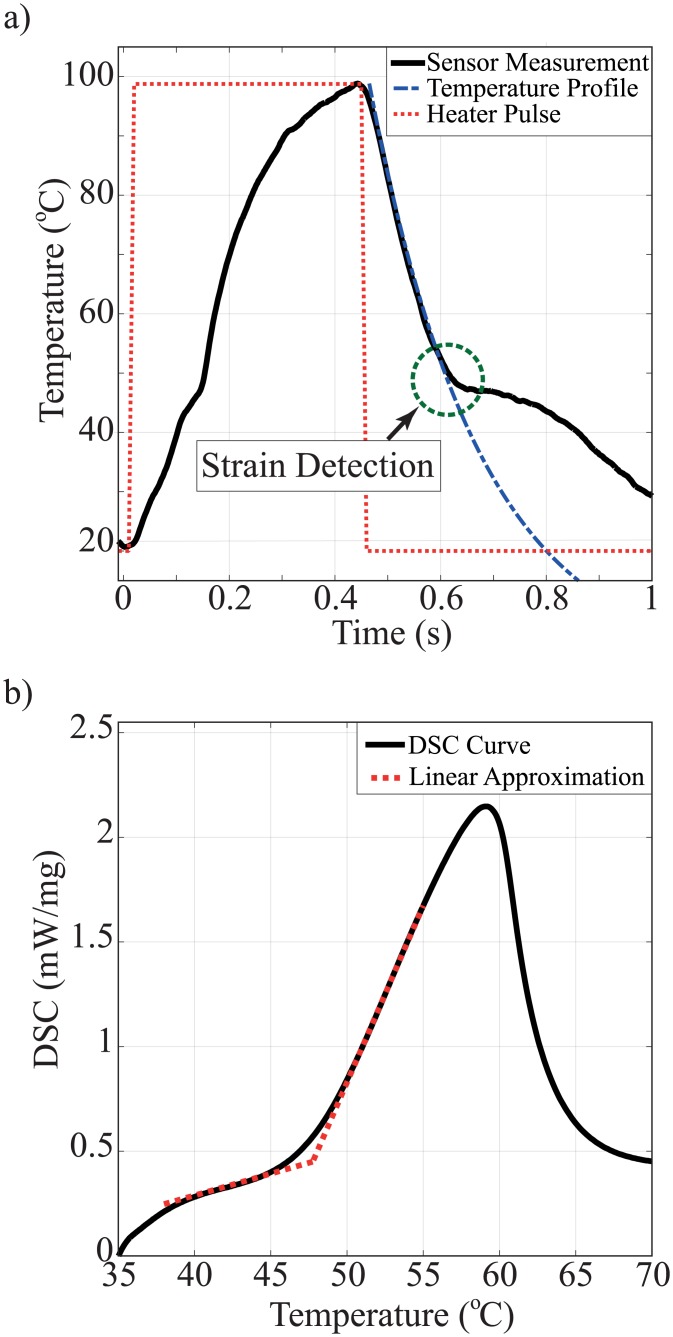
Phase transition measurement of purified wax. (a) Micro-thermomechanical measurement by sensor #1. The transition temperature is detected during either the heating or the cooling cycle. The deviation point is circled. The deviation is caused by the stress applied by the sample during the phase transition. (b) Standard DSC method for the phase transition measurement. The linear approximation is used to determine the phase transition temperature.

The operation of the experimental setup and accuracy of the sensor are demonstrated by measuring the phase transition temperature of a purified wax and comparing with the standard DSC measurement result. The sample size for our method and DSC are 50 *μg* and 10 *mg*, respectively. Fig 4 shows the temperature profile of the wax sample measured by the thermomechanical calorimeter system during heating and cooling from room temperature to 100°*C*. The temperature response of the sensor is interrupted by mechanical strain at the phase transition temperature of the wax as shown in Fig 4(a). The dotted line in the cooling cycle is the temperature profile of the wax sample when no mechanical strain is applied to the sensor. The temperature profile change is more pronounced at the cooling cycle than at the heating cycle. The difference is currently under investigation. The phase transition temperature of the wax is measured by a commercial DSC as shown in Fig 4(b). The micro-thermomechanical method and the DSC show the phase transition of the wax as 48.2°*C* and 47.8°*C*, respectively. The result shows the phase transition temperature measured by the micro-thermomechanical method agrees with the DSC method.

### Sol-gel and gel-sol transition detection

**Fig 5:**
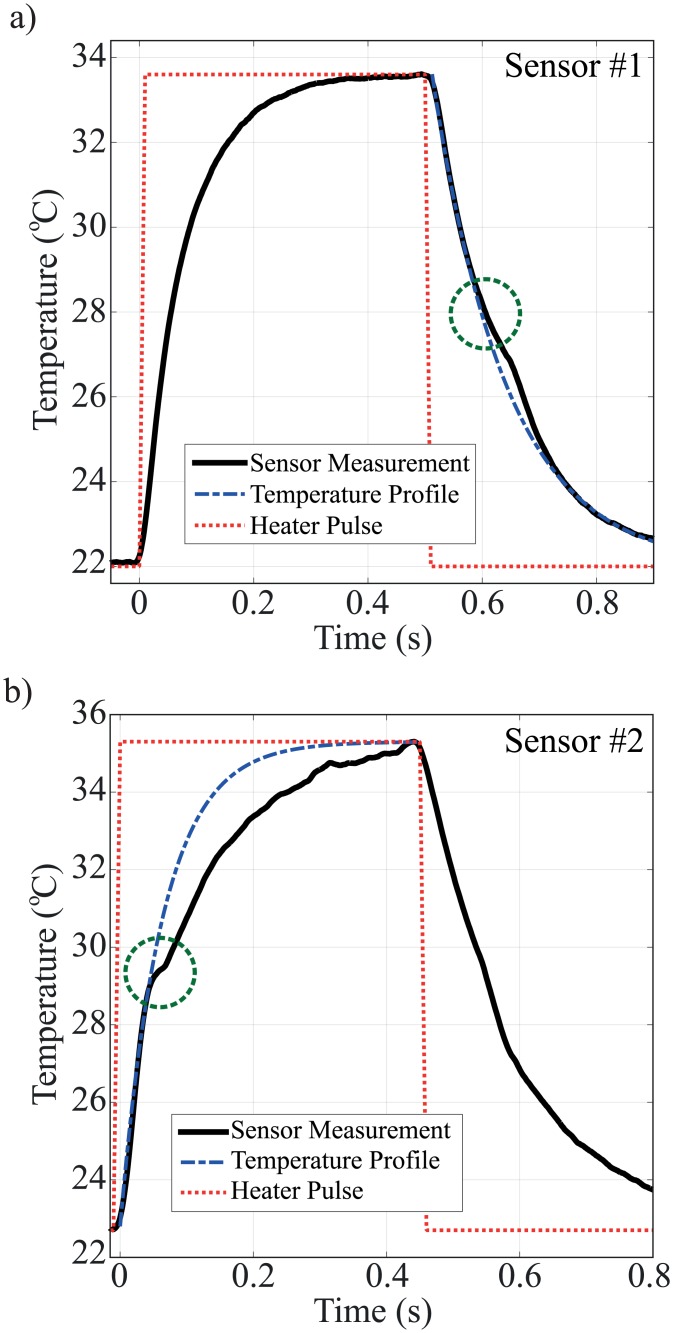
Thermomechanical method for gelation temperature detection of 15% gelatin, measured by sensor integrated on a) 500 *μm* by 500 *μm* (sensor #1), and b) 5 *mm* by 5 *mm* (sensor #2) SiN membranes. The temperature profile represents the first order approximation of heat transfer through the material. The gelation temperature is measured during either heating or cooling cycle depending on the sensor type. The gelation occurs when the measurement deviates from the temperature profile due to strain applied to the sensor.

The gelation temperature of different concentrations of gelatin is measured by the same technique. The samples are heated past their known gelation temperatures, measured by rheology, by applying 4-6 *mW* power to the heater. Fig 5(a) shows the gel-sol transition of 15% gelatin detected by sensor #1 during cooling. The mechanical strain is caused by the volume shrinkage during sol-gel transition as a results of self-assembly of gelatin chains and increases the resistance of the sensor. Fig 5(b) shows the sol-gel transition of 15% gelatin detected by sensor #2 during a heating cycle. The volume expansion during melting causes a compression strain on the membrane and decreases the resistance.

The only difference between sensor #1 and sensor #2 is the size of the membrane. The membrane size of sensor #1 (500 *μm* ×500 *μm*) is smaller than that of sensor #2 (5 *mm* ×5 *mm*). Fig 5 clearly shows that the resistance changes due to the strain at the phase transition of the hydrogel are detectible during the cooling and heating cycle. Although the mechanisms of increasing or decreasing resistance due to the compressive or tensile strain are well understood, the resistance change observed during the cooling or heating cycle alone is still under investigation and is discussed in discussion section.

The gelation temperatures of 10, 15, and 20 wt% gelatin solutions are measured by both sensor #1 and #2. Sensor #1 measures the cooling (sol-gel) phase transition and Sensor #2 measures the heating (gel-sol) phase transition.

**Fig 6:**
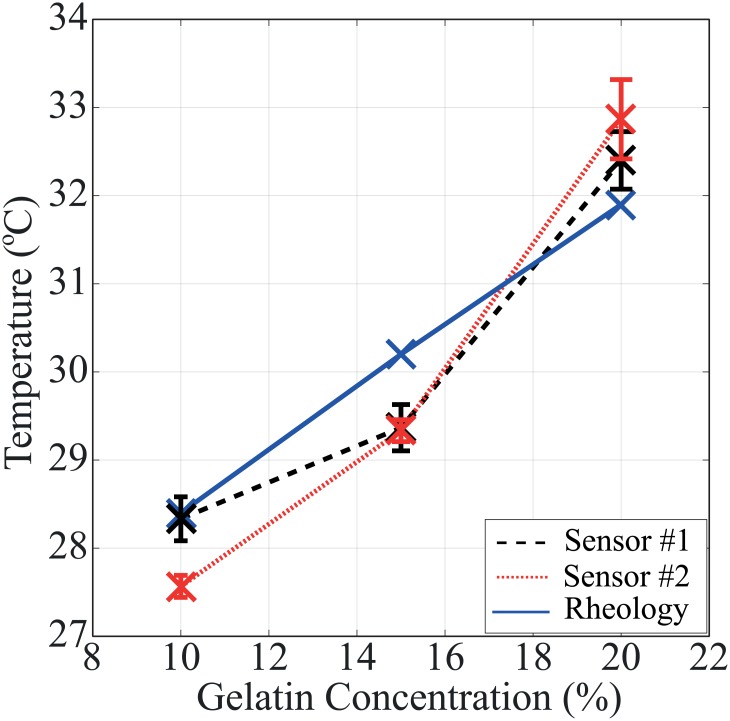
Gelation temperatures of 10%, 15%, and 20% gelatin measured by the micro-thermomechanical method using sensor #1 (dashed line), sensor #2 (dotted line), and standard rheology (solid line).

The results are compared with the standard rheology phase transition measurement of bulk samples as shown in Fig 6. The results are summarized in Table 1. The micro-thermomechanical measurement method shows less than a degree Celsius of deviation and 3% error from the rheology results.

**Table 1:** Gelation temperatures of 10%, 15%, and 20% gelatin, measured by rheology and micro-thermomechanical sensors for comparison.

		Sensor#1		Sensor#2	
Gelation Conc. (wt%)	Rheology (°*C*)	Average (°*C*)	Error (%)	Average (°*C*)	Error (%)
10	28.4	28.33±0.25	0.25	25.57±0.12	2.92
15	30.2	29.37±0.26	2.75	29.33±0.12	2.88
20	31.9	32.40±0.33	1.57	32.87±0.45	3.04

## Discussion

### Selective detection of sol-gel and gel-sol transitions

The resistance change due to the strain at the transition temperature is observed during the heating or cooling cycle depending on the sensor. The selective detection of sol-gel and gel-sol transitions of gelatin by sensors #1 and #2, respectively, are likely due to the amount of gelatin that goes under the phase transition. The disk area of the 1 *μl* gelatin sample is 5 *mm*^2^ while the membranes of sensors #1 and #2 are 0.25 *mm*^2^ and 25 *mm*^2^. Therefore, all of sensor #1 and 20% of sensor #2 membranes are in contact with the gelatin. During the heating cycle, the thermal expansion of the gelatin can be seen through the microscope camera. The gelatin hydrogel on the heater membrane expands upon melting, and the gelatin solution flows away from the heater.

For sensor #1, during the heating cycle, the volumetric expansion of the gelatin applies a relatively small strain to the membrane because the solution between the membranes flows sideways. Because the gelatine flows, no strain is applied to the membrane. During cooling, however, the volume collapses steadily, pulling the membrane down causing a tensile stress on the membrane because the entire membrane is in contact with the shrinking gelatin. The effect of the tensile stress on the membrane is an additional resistance increase as shown in Fig 5(a).

For sensor #2, the membrane is much larger than the sample. The edge of the sample is located far away from the edge of the membrane, where the most sensitive sensor part to the strain is located. When the gelatin solution melts, it likely spreads toward the edge of the membrane, applying a compressive stress to the sensor membrane. This compressive stress results in a decreasing resistance as shown in Fig 5(b). Although the sample shrinks during the cooling cycle, no significant tensile stress is applied to the large membrane because only 20% of the sensor #2 membrane is in contact with the shrinking gelatin.

### Defining the phase transition points

In the thermomechanical method, the phase transition temperature is determined by identifying the deviation point from the cooling temperature profile (dashed line) as shown in Fig 4(a). The deviation is caused by the strain of the sample at the transition temperature.

For the wax sample, the transition is easily detectable both in the thermomechanical method and the DSC. In the thermomechanical method, the strain on the membrane is relatively large at the phase transition. The deviation is distinct and the transition temperature can be fairly easily determined. Since the wax sample has relatively high enthalpy change (180 *J*/*g*), the phase transition is clearly detected from the temperature slope in the DSC method as well [35].

On the other hand, the stress from the gelatin transitions is significantly lower and affects the resistance change less than the wax as shown in Fig 5(a). Even in the low stress (low volume change) condition at the phase transition, the sensor can be modified to amplify the strain signal. A larger membrane can be used to detect the phase transition point during the heating cycle as shown in Fig 5(b). The resistance change without the strain effect can be fitted to the first order approximation as shown in Fig 5 (dashed line). The deviation point from the fitted curve indicates the temperature at which the sensor starts experiencing the strain.

The sol-gel transition enthalpy of hydrogels are significantly lower compared to the wax. The transition enthalpy of gelatin gel is measured to be 15-30 *J*/*g* [36]. Therefore, it has been a challenge to measure the transition point with a DSC. The transition temperature of hydrogels is typically measured by a rheology method. Even in the case of low enthalpy change and low stress of hydrogel at the phase transition, the thermomechanical method can measure the phase transition temperature.

The gel-sol and sol-gel transition temperatures of gelatin at microscale are in close agreement with the macroscale sol-gel transition temperature measured by rheology method. Previous studies show that the gelatin transition temperatures could differ up to 5°*C* depending on the thermal history of the material [8, 36]. Heat tends to break gelatin’s inter- and intramolecular bonds into their lowest energy configurations, changing the gel structure. Therefore, the thermal history of gelatin affects its thermal properties, including the gelation temperature. For example, the properties of gelatin cooled down to 25°*C* from 40°*C* are different than the properties of gelatin heated from 10°*C* to 25°*C* [8]. However, in our study, the transition temperatures acquired by different sensors during heating and cooling cycles is less than half a degree Celsius of difference. The error of the sol-gel phase transition temperature measurement may be reduced by keeping parameters such as the storing temperature and time of the samples consistent.

## Conclusion

A newly developed micro-thermomechanical method to measure the phase transition temperature of physical hydrogel systems at microscale is presented. The thermomechanical detection mechanisms and operation principle are discussed in detail. The new method can measure the minute volume expansion and contraction of microscale samples at the gel-sol and sol-gel transition temperatures using a piezoresistive RTD sensor integrated on a membrane. We introduce a unique device configuration to measure physical and chemical transformations of gel/solid at a microscale volume that is not possible with typical microfluidic systems. The gelation temperature of various concentrations of a hydrogel measured by the micro-thermomechanical method agrees with the results from the rheology method at less than 3% of error. The micro-thermomechanical method can detect the phase transition of low enthalpy change materials, which may be challenging with a typical micro-DSC.
